# The Role of CD4/6 Inhibitors in Breast Cancer Treatment

**DOI:** 10.3390/ijms25021242

**Published:** 2024-01-19

**Authors:** Luv Purohit, Can Jones, Teresita Gonzalez, Aurelio Castrellon, Atif Hussein

**Affiliations:** Memorial Health System, Pembroke Pines, FL 33024, USA; luvpurohit@gmail.com (L.P.); canxjones@mhs.net (C.J.); tergonzalez@mhs.net (T.G.); ahussein@mhs.net (A.H.)

**Keywords:** CDK inhibitors, ER+/HER2− breast cancer, ctDNA

## Abstract

Over the last decade, treatment paradigms for breast cancer have undergone a renaissance, particularly in hormone-receptor-positive/HER2-negative breast cancer. These revolutionary therapies are based on the selective targeting of aberrancies within the cell cycle. This shift towards targeted therapies has also changed the landscape of disease monitoring. In this article, we will review the fundamentals of cell cycle progression in the context of the new cyclin-dependent kinase inhibitors. In addition to discussing the currently approved cyclin-dependent kinase inhibitors for breast cancer, we will explore the ongoing development and search for predictive biomarkers and modalities to monitor treatment.

## 1. Introduction

Breast cancer, the most prevalent cancer among women, is a heterogeneous disease with various subtypes. The most common subtype, hormone-receptor-positive/HER2-negative (HR+ or ER+/HER2−), accounts for 69% of all cases [1]. This subtype, like others, proliferates through dysregulation of the cell cycle, particularly the transition from the G1 to S phase, facilitated by cyclins and cyclin-dependent kinases (CDKs) [2]. CDK 4/6 inhibitors, such as palbociclib, ribociclib, and abemaciclib, have emerged as innovative therapeutics for advanced or metastatic HR+/HER2− breast cancer. These drugs target the disrupted pathway in breast cancer and have shown significant efficacy in improving progression-free and overall survival, especially when combined with hormone therapy [3,4,5,6,7,8,9,10,11]. Despite their similar mechanism of action, these inhibitors exhibit nuanced pharmacological differences, affecting their clinical utility [12]. Currently, the optimal sequencing of these therapies with other treatments, like endocrine therapy, is an area of ongoing research. The key to determining the appropriate sequence of therapy lies, in part, in finding predictive biomarkers to determine the response to CDK 4/6 inhibitors. Such biomarkers could enable a more tailored therapeutic approach, potentially improving response rates and minimizing unnecessary toxicity. This review will discuss the basic science behind CDK inhibitors, the seminal trials which led to the approval of these inhibitors in HR+/HER2− breast cancer, their use in the adjuvant setting as well as in HR+/HER2+ breast cancer, and the ongoing research to determine optimal sequencing and find predictive biomarkers.

## 2. CDK Pathway and Inhibition

### 2.1. The Pathway

Cyclin-dependent kinases interact with the E2F transcription factor and retinoblastoma (Rb) protein in a pathway essential to the regulation of the cell cycle. It involves the interaction CDK4 and CDK6 with D-type cyclins to form an inactive ternary complex. The complex is stabilized and transported to the nucleus by proteins p21 and/or p27. Interestingly, p21 and p27 also serve as CDK inhibitors by sequestering CDK 4/6, thereby serving to both facilitate activation and directly inhibit the cyclin-CDK4/6 complex [13].

**Figure 1:**
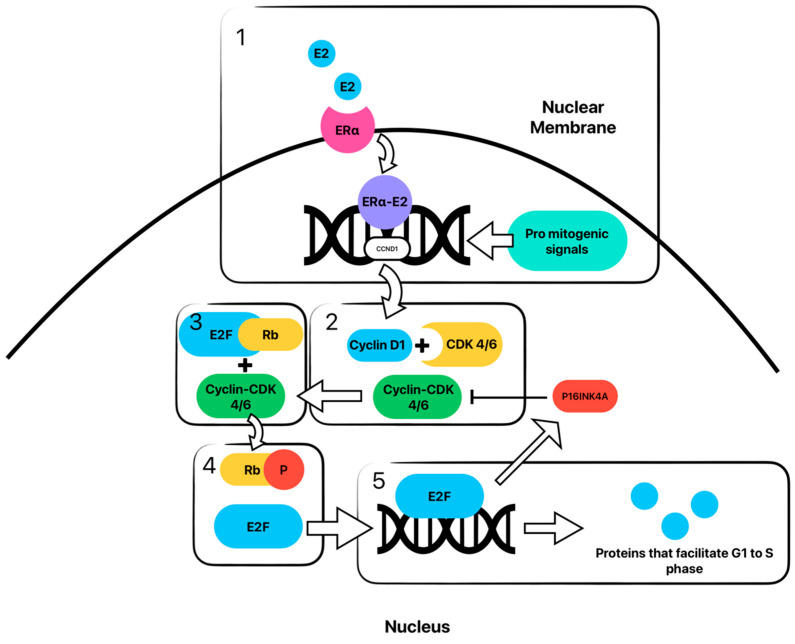
Cyclin-CDK-E2F-Rb pathway with estrogen signaling resulting in amplification of cyclin D is shown. In **box 1**, estrogen (E2) binds with estrogen receptor (ER) and is translocated to the nucleus, where it, then promotes the transcription of the CCND1 gene resulting in cyclin D1. Pro-mitogenic signals and other pathways can also amplify cyclin D1 transcription, which is discussed elsewhere. In **box 2**, cyclin D1 then binds with cyclin-dependent kinase (CDK) 4 and 6 to create an activated holoenzyme. In **box 3**, this cyclin-CDK 4/6 complex phosphorylates retinoblastoma protein (Rb), which results in the dissociation of Rb from the E2F transcription factor seen in **box 4**. In **box 5**, the now free E2F transcription factor promotes the downstream cascade of protein synthesis, ultimately transitioning the cell from G1 to S phase. Further, E2F transcribes the CDK2NA gene, which results in the synthesis of p16INK4a, a potent inhibitor of the cyclin-CDK 4/6 complex. This results in a negative feedback loop, which arrests the progression of the cell cycle.

Once the cyclin-CDK4/6 complex is translocated to the nucleus by p21/p27, it is phosphorylated at T172 by cyclin activating kinase (CAK). This phosphorylation has been shown to be the rate-limiting step of cyclin-CDK4/6 complex activation. The now active and stabilized holoenzyme then phosphorylates the Rb protein, resulting in the inactivation of Rb. Once Rb is inactivated, the E2F transcription factor is released, resulting in the transcription of genes responsible for the transition of the cell from the G1 to the S phase [14]. This is illustrated in Figure 1.

One specific gene that is transcribed because of this process is the CCNE1 gene, which, when transcribed, forms cyclin E. In an interesting positive feedback loop, cyclin E binds with cyclin-dependent kinase 2 (CDK2), which in turn hyperphosphorylates Rb, resulting in further suppression of Rb [15].

Ultimately, the release of E2F through the phosphorylation of Rb by CDK–cyclin complexes results in the progression of the cell from the G1 to the S phase.

### 2.2. Regulation of the Pathway

D-type cyclins (D1, D2, and D3) are produced by the gene CCND1 in response to various external signals, including growth-promoting mitogens, inhibitory cytokines, and differentiation signals, amongst others. It is worth noting that CCND1 is a transcriptional target of nuclear receptors such as the estrogen receptor (ER) [16]. This supports why ER-positive breast cancer patients respond well to the therapeutic combination of CDK 4/6 inhibition and endocrine therapy, as there is an overexpression of cyclin D via ER activation.

Following the discovery of D-type cyclins, CDK4 and CDK6 were identified. These kinases were found to bind to and be activated by any of the three D-type cyclins. CDK4 and CDK6 function during the G1 phase to push quiescent cells that have entered the cell cycle, or proliferating cells that have completed mitosis, toward the S phase [17]. This process is regulated by the CDKN2A gene, which produces p16INK4a, an inhibitor of the CDK4/6 enzyme. It is worth noting that mutations in CDK2NA can result in the loss of p16INK4a, resulting in unchecked progression from the G1 to the S phase [18].

### 2.3. Inhibition of the Dysregulated Pathway

The CDK E2F Rb pathway has been a long-sought-after target for cancer therapy, as it is dysregulated in virtually all human cancer cells [19]. Dysregulation can occur through one of many processes, including the overexpression of cyclin D1, the loss of endogenous CDK inhibition vis-a-vis the absence of p16INK4a, the mutation of CDK4 to a p16INK4a-refractory state, or the loss of the Rb1 gene [20]. This dysregulation results in unfettered cellular proliferation, forming the basis of tumorigenesis. The inhibition of this pathway and preventing the phosphorylation of Rb halts the cell cycle in the G1 phase, effectively inducing cell cycle arrest or cytostasis [20]. As a result, these inhibitors can curtail the uncontrolled proliferation of cancer cells.

The initial CDK inhibitors were pan-selective and ultimately ineffective due to their significant dose-limiting toxicities. Agents such as flavopiridol and roscovitine inhibited CDK 1, 2, 4, 6, 7, and 9, resulting in cellular cytotoxicity [21,22]. A trio of new CDK inhibitors, palbociclib, ribociclib, and abemaciclib, are highly selective for CDK4/6, resulting in potent cytostatic activity. However, variance in their chemical structures results in differences in their pharmacology [23].

Palbociclib and ribociclib are the closest in chemical structure and thus exhibit similar potency in CD4 inhibition; however, palbociclib inhibits CD6 more potently than ribociclib [3,6]. They also share similar toxicity profiles, with myelosuppression being the dose-limiting toxicity. A key differentiator between these two agents is the prevalence of QT prolongation with ribociclib. As such, patients with co-morbid cardiac conditions are advised to avoid ribociclib [8]. These two agents are also dosed once daily due to their long half-lives.

The chemical structure of abemaciclib varies from its siblings in several key ways and may explain its unique biochemical and clinical properties [24]. Abemaciclib has several unique interactions with CD6. For one, it is the only CDK inhibitor that can form a hydrogen bond with CD6 [24]. Further, due to its structure and lipophilicity, it can access deep within the ATP-binding pocket of CD6, where ribociclib and palbociclib cannot [24]. The result of this is a highly potent inhibition, which can be observed when comparing the IC50s between the three agents [24].

Although a potent CDK4/6 inhibitor, abemaciclib also has an inhibitory activity on cyclin T1/CDK9, cyclin E2/CDK2, p25/CDK5, and p35/CDK5. This point is crucial in understanding why abemaciclib is the only agent efficacious as a monotherapy, as determined by the MONARCH-3 trial [11,24]. A proposed mechanism for CDK4 inhibition resistance is the upregulation of cyclin E2/CDK2 function, which essentially takes over the role of cyclin D-CDK4/6 in the cell progression from the G1 to the S phase [25]. Abemaciclib’s inhibitory activity against E2/CDK2 could hamper this compensatory pathway. Additionally, early agents like flavopiridol and roscovitine were potent inhibitors of CDK9, a key player in global transcription regulation [21,22]. The inhibition of CDK9 results in cellular cytotoxicity, suggesting that abemaciclib also exhibits a cytotoxic effect in addition to the cytostatic effects of CDK4/6 inhibition [26]. This relative “pan-selectivity” of abemaciclib compared to the other CDK inhibitors could also, at least in part, explain its unique GI toxicity profile [11]. Abemaciclib has a shorter half-life and, thus, is dosed continuously and twice daily compared to the once-daily dosing of palbociclib and ribociclib [11]. The difference in chemical structures of the three agents can be compared in Table 1. 

## 3. Clinical Use of CDK 4/6 Inhibitors

### 3.1. Metastatic or Advanced ER+/HER2− Breast Cancer

The first CDK4/6 inhibitor approved for first-line treatment for ER+/HER2− breast cancer treatment was palbociclib after the pivotal PALOMA-1 trial in 2017 [3]. The PALOMA-1 trial, a phase II study, involved 165 treatment-naive postmenopausal patients. It found that palbociclib, when combined with letrozole, improved progression-free survival (PFS) from 10.2 to 20.2 months.

The PALOMA-2 trial, a phase III study, enrolled 666 similar patients and found that palbociclib improved PFS from 14.5 to 24.8 months when added to letrozole [4]. The PALOMA-3 trial, another phase III study, involved 521 women who had progressed on previous endocrine therapy. It was found that palbociclib prolonged PFS from 4.6 to 9.5 months and overall survival when added to fulvestrant [5].

All PALOMA trials showed palbociclib’s PFS improvement when added to endocrine therapy and its good tolerability, with neutropenia being the most common side effect.

Today, palbociclib is the most widely used agent in combination therapy with ET for advanced ER+/HER2− breast cancer. Approval for palbociclib was followed shortly after by the approval of ribociclib and abemaciclib through the MONALEESA-2 and MONARCH-2 trials, respectively [6,9].

The MONALEESA clinical trial program investigated the clinical activity and safety of ribociclib. The program included three trials: MONALEESA-2, MONALEESA-3, and MONALEESA-7.

The MONALEESA-2 trial evaluated the combination of ribociclib with letrozole as a first-line therapy in postmenopausal women with advanced breast cancer (ABC). The median progression-free survival (PFS) in patients who received ribociclib was 25.3 months versus 16 months in those who received a placebo (hazard ratio (HR): 0.568 (95% CI: 0.457–0.704); *p* < 0.001) [6].

The MONALEESA-3 trial evaluated ribociclib plus fulvestrant in postmenopausal women who had relapsed >12 months from their endocrine therapy or presented with de novo ABC. The median PFS was 20.5 months (95% CI: 18.5−23.5 months) and 12.8 months in the ribociclib and placebo arm, respectively (HR, 0.593, 95% CI, 0.480–0.732; *p* < 0.001) [7].

The MONALEESA-7 trial evaluated a treatment of ribociclib plus goserelin with either tamoxifen or a nonsteroidal aromatase inhibitor (NSAI) in pre/perimenopausal women. The median PFS was 23.8 months in the ribociclib group and 13.0 months in the placebo group (HR, 0.55, 95% CI, 0.44–0.69; *p* < 0.0001) [8].

The overall safety profiles of ribociclib in all three trials were similar. The most common adverse events of any grade that occurred in ≥25% of patients were neutropenia, leukopenia, and nausea. Corrected QT interval (Fridericia’s formula) prolongation was reported in <10% of patients receiving ribociclib in each of the three trials [6,7,8].

Abemaciclib received its first approval from the U.S. Food and Drug Administration (FDA) in September 2017 [8]. It was initially approved for the treatment of HR-positive, HER2-negative advanced or metastatic breast cancer. Since then, its approved use has expanded to include its utilization as a monotherapy [9,10,11].

The MONARCH 2 trial focused on patients who had progressed during prior endocrine therapy. It involved 669 women, with 446 randomized to the abemaciclib-plus-fulvestrant arm and 223 to the placebo-plus-fulvestrant arm. The trial found a statistically significant improvement in overall survival (OS) in the abemaciclib group, with a median OS of 46.7 months compared to 37.3 months in the placebo group (HR, 0.757, 95% CI, 0.606–0.945; *p* 0.014). The improvement was more pronounced in patients with visceral disease and those with primary resistance to prior endocrine therapy. Other measures such as time to second disease progression, time to chemotherapy, and chemotherapy-free survival were also significantly improved in the abemaciclib group [10].

The MONARCH-3 trial evaluated abemaciclib as an initial treatment for postmenopausal women with HR+, HER2− advanced breast cancer. The trial involved 493 women and found that the median progression-free survival (PFS) was significantly longer in the abemaciclib group (28.18 months) compared to the placebo group (14.76 months). The objective response rate (ORR) was also higher in the abemaciclib group (61.0% versus 45.5%) [11].

In both trials, the safety profile of abemaciclib was consistent with previous reports, with the most frequent grade ≥3 adverse events being neutropenia, diarrhea, and leukopenia [10,11].

All three trial programs, PALOMA (1, 2, 3), MONALEESA (2, 3, 7), and MONARCH (1, 2, 3), showed significant improvement in PFS amongst advanced ER+/HER2− breast cancer patients across all menopausal statuses; however, there were some key differences.

Overall survival was only seen with ribociclib in the MONALEESA-2 and -3 trials, whereas the PALOMA trials did not show a significant increase in OS [3,4,5,6,7]. It is unclear why this is. It can be speculated that this could be due to differences in CDK inhibition potency and missing survival data from the PALOMA trial; however, without a head-to-head study, conclusions remain speculation. The data for OS with abemaciclib is still maturing; however, interim analyses do show a trend suggesting that the final analysis will result in an improvement in OS [27].

Among the three drugs, only abemaciclib has been approved as a monotherapy after the results of the MONARCH-1 phase 2 trial, which studied heavily pre-treated ER+/HER2− breast cancer patients who progressed on or after ET and chemotherapy. They found that patients on continuous monotherapy with abemaciclib had an ORR of 19.7%, a CBR of 42.4%, an mPFS of 6 months, and an OS of ~18 months [9]. The trials referenced above are summarized in Table 2. 

### 3.2. Considering Endocrine Resistance in Treatment of ER+/HER2− Advanced Breast Cancer

There are many patients that do well with endocrine therapy alone; however, there are some that progress. Approximately 1 in 6 women with node-positive HR+/HER2− early-stage BC receiving endocrine therapy experience recurrence or death within 5 years of initiating treatment [28]. However, most recurrences occur after 5 years, a phenomenon that is largely attributed to endocrine resistance [28]. Endocrine resistance is a heterogeneic process consisting of multiple potential resistant pathways. The most common process is the loss of ESR1 gene expression through CpG island methylation or histone deacetylase activity on the ESR1 promoter [29]. The ESR1 gene normally transcribes the estrogen receptor, so the loss of ESR1 results in resistance to anti-estrogen therapy [29]. Other processes include the up-regulation of the MAPK and PI3K pathways, which results in the estrogen-independent activation of the ER pathway and the over-expression of cyclin D [30]. These pathways act upstream of the cyclin-CDK-E2F pathway and thus suggest why the new CDKis have shown PFS in patients who progressed on or after ET. The advent of CDK4/6 inhibition has been groundbreaking, as it is a common pathway for several resistance mechanisms; however, resistance develops with CDK inhibition as well, underscoring the complexity of endocrine resistance.

Currently, combination treatments, including the inhibition of other pathways such as P13K, are currently being studied. Alpelisib, a PI3K inhibitor, was approved by the FDA in 2019 for use in combination with fulvestrant for the treatment of PIK3CA-mutated, hormone-receptor-positive, HER2-negative advanced or metastatic breast cancer [31].

### 3.3. Determining Sequence of Therapy

Given that CDKI’s are approved for first- and second-line therapy, endocrine resistance is inevitable with or without CDK inhibition. Since many patients do well on endocrine monotherapy, the placement of the new CDK inhibitors in the therapeutic lineup becomes challenging.

The SONIA trial, recently published in 2023, evaluated the efficacy, safety, and cost-effectiveness of CDK4/6i added to either first- or second-line endocrine therapy (ET) in patients with HR+, HER2− ABC who had received no prior therapy for ABC [32]. In the trial, 1050 pre- and post-menopausal woman were randomized to either strategy A or B. Strategy A consisted of a first-line CDKi + non-steroidal aromatase inhibitor with progression treated with fulvestrant. Strategy B consisted of a first-line NSAI with progression treated by CDKi + fulvestrant. Which CDKi to use was determined by the treating physician; however, over 90% used palbociclib.

The study found no statistical difference in their primary endpoint of PFS between either strategy. The median PFS2 was 31.0 months in strategy A versus 27.8 months in strategy B (hazard ratio: 0.89; 95% confidence interval: 0.75 to 1.04; *p* = 0.14). Secondary endpoints such as toxicity and cost-effectiveness were also examined, which determined that first-line use prolonged the time on CDK4/6i by 16.4 months and increased toxicity and costs [32].

This study suggests that although CDKi’s are approved for both first- and second-line treatment of ABC, it may be reasonable to reserve their use for the second-line setting to avoid both drug and financial toxicity. There is speculation that PFS was blunted due to the use of fulvestrant monotherapy in the second line, a treatment strategy that is uncommon in today’s practice. The results of this study underscore the importance of tailoring treatment using predictive biomarkers.

### 3.4. Adjuvant Use of CDK4/6 Inhibitor in Early Breast Cancer (EBC): PENELOPE-B, PALLAS, MonarchE, and NATALEE Trials

CDK4/6 inhibitors have been implemented as an adjuvant treatment for EBC. Specifically, two CDK4/6 inhibitors are becoming part of the standard treatment for HR+, HER2− EBC. Two main studies have been pivotal in achieving this accomplishment: MonarchE and NATALEE. The MonarchE study paved way for the utilization of adjuvant abemaciclib with endocrine therapy (ET) in the setting of high-risk EBC, while the NATALEE trial data presented at ASCO 2023 added ribociclib to the landscape of adjuvant CDK4/6 inhibitors in a similar setting.

Before these trials emerged, the PENELOPE-B trial was a randomized, double- blind, phase III trial that investigated the use of palbociclib (PAL) in HR+, HER2− breast cancer patients with residual disease after neoadjuvant chemotherapy who were at high risk of relapse [33]. A total of 1250 women were enrolled in the trial. In one arm, PAL was given for 13 cycles with at least 5 years of ET compared to ET with placebo. Unfortunately, this trial failed to meet its primary endpoint of improved invasive disease-free survival (iDFS). However, this was the first trial that had mature data on the use of a CDK4/6 inhibitor as part of adjuvant treatment for early breast cancer, paving the way for more studies in the non-metastatic setting.

Similarly, in a prospective, randomized, phase III PALLAS trial investigating the use of PAL in HR+ EBC, there was no statistically significant difference between either treatment arm. At a median follow-up of 31 months, iDFS occurred in 8.8% in the PAL + ET group compared to 9.1% in the ET alone arm, with an iDFS at 4 years of 84.2% vs. 84.5% (HR = 0.96, 95% CI: 0.81–1.14, *p* = 0.65) [34]. In 2021, abemaciclib gained approval in the treatment of high-risk HR+, HER2−, node-positive breast cancer patients based on the data from the MonarchE study. The MonarchE study enrolled patients with HR+, HER2− high-risk EBC [34]. High risk was defined as ≥4 axillary lymph nodes (ALN) or 1–3 ALN with either tumor size ≥ 5 cm, histologic grade 3, or Ki67 ≥ 20%. One arm received abemaciclib (150 mg twice daily for 2 years) with ET for 5–10 years, and the other arm received ET alone. The primary endpoint was iDFS. Overall, the 3-year iDFS improved by 5.4% in the abemaciclib group (HR = 0.70, 95% CI: 0.59–0.82), with the primary toxicity being diarrhea and neutropenia. This study was groundbreaking, in that it was the first study to demonstrate an improvement in iDFS with the use of a CDK4/5 inhibitor in the adjuvant setting in patients with HR+, HER2− EBC at risk of relapse [35]. The NATALEE trial, presented at ASCO 2023, is an ongoing, open-label, large, randomized, multicenter, phase 3 trial investigating the use of adjuvant ribociclib with ET in stage II and III patients with HR+, HER2− breast cancer [36]. Out of a total of 5101 patients, 2549 patients were randomized 1:1 to receive either ribociclib and ET ≥ 5 years (with either letrazole or anastrozole) (RIBO + ET) or ET only (2552 patients). Ribociclib was dosed at 400 mg/day, 3 weeks on, 1 week off, for 3 years. The primary endpoint was iDFS. The secondary endpoints included recurrence-free survival (RFS), overall survival (OS), distant-disease-free survival (DDFS), safety, and tolerability. The absolute iDFS rate difference was 3.3%, favoring the ribociclib group, with a risk reduction of 25.2%. The absolute distant-disease-free survival difference was 2.2% favoring ribociclib, with a 26.1% reduction in distant disease risk. About 20% of the patients completed 3 years of ribociclib, with 19% discontinuing due to adverse events. The most frequent adverse events that led to discontinuation included elevation in liver function tests and arthralgia. Grade 3 or higher neutropenia and QTc prolongation was also reported in the ribociclib arm.

This study was a breakthrough, as it was large, including more patients than the MonarchE study. It was different from previous studies in that it included patients with no nodal involvement (N0) with high-risk features, setting a new standard in N0 patients. A lower dose of ribociclib was utilized (400 mg instead of 600 mg) compared to the MONALEESA trial, which allowed for a higher tolerability. It also extended the duration of CDK4/6 inhibitor use to 3 years instead of the previously studied 2 years with abemiciclib in the MonarchE study. One of the limitations was that not all the patients in the high-risk N0 group received standard chemotherapy, favoring the ribociclib arm. Moreover, with an absolute iDFS rate difference of 3.3%, the standards to better define minimal clinically relevant differences should be further explored. Overall, this is an impressive study that will likely reshape the standard of care in high-risk EBC patients. It would be interesting to see the long-term survival data for this study in the future. The trials referenced above are summarized in Table 3.

### 3.5. CDK Inhibtor Use in ER+/HER2+ Breast Cancer

CDK 4/6 with cyclins plays a critical role in cell proliferation. The ER and HER2 pathways, independently or collectively, converge to facilitate cell proliferation. HER2 signaling targets cyclin D downstream. Preclinical studies have shown that ER+/HER2+ breast cancer is responsive to cell cycle inhibitors [37]. As a result, there have been emerging studies working on treating ER+/HER2+ disease with a combination of CDKi and HER2 target therapy. It may provide synergistic pharmacological benefits in limiting tumor progression, enhancing treatment sensitivity, and improving mortality [38].

The SOLTI-1303 PATRICIA trial is a prospective, randomized, open-label, multicenter, phase II trial. It was designed to evaluate the efficacy and safety of palbociclib in combination with trastuzumab with or without letrozole in treating localized advanced or metastatic ER+/HER2+ breast cancer patients who had received 2–4 prior lines of anti-HER2-based regimens. Seventy-one postmenopausal patients were divided into three cohorts based on the ER status: ER- (cohort A), ER+ (cohort B1), and ER+ with letrozole (cohort B2). The primary interest was the PFS rate at 6 months. The secondary objectives included the safety and evaluation of the PAM50 intrinsic subtypes [39]. 

The study revealed that the PFS rate at 6 months in cohorts A, B1, and B2 was 33.3% (5/15), 42.8% (12/28), and 46.4% (13/28), respectively. The safety analysis showed that 97.7% of patients had grade 1–2 toxicities and 84.4% of patients developed 3–4 toxicities including neutropenia (66.4%) and thrombocytopenia (11.3%). Luminal disease defined by PAM50 was reported to be associated with a longer PFS compared with nonluminal disease (PFS: 10.6 vs. 4.2 months; adjusted hazard ratio: 0.40; *p* = 0.003). In conclusion, palbociclib used with trastuzumab is safe and shows survival benefits in ER+/HER2+ advanced breast cancer with a PAM50 luminal disease [39].

The PATINA trial is a randomized, open-label, international, phase III trial. It was designed to evaluate the safety and efficacy of palbociclib in combination with anti-HER-2 and endocrine therapy vs. anti-HER2 and endocrine therapy in HR+/HER2+ metastatic breast cancer patients [40]. After receiving 4–8 cycles of induction chemotherapy (taxane or vinorelbine) with an anti-HER2 regimen, 496 patients were randomized to receive anti-HER2-plus-endocrine therapy, with or without palbociclib. The primary interest is the PFS. The secondary outcomes included OS, 3- and 5-year survival probabilities, the objective response rate (OR), the clinical benefit rate, safety, patient-reported outcomes, and the incidence of CNS metastasis. The trial is being conducted in Australia, New Zealand, the United States, Spain, and Germany. The study hypothesized that the addition of a CDKi to anti-HER2 and endocrine therapy after induction therapy would delay the onset of therapeutic resistance, prolong survival, and improve endocrine resistance in HR+/HER2 breast cancer [40]. The trials referenced above are summarized in Table 4. 

## 4. Search for Biomarkers

With the results of the SONIA trial and the inevitable development of resistance, when to use CDK inhibition becomes an important question. The key to answering this question lies in predictive biomarkers that can identify de novo and acquired CDK inhibitor and ET resistance mechanisms. Such biomarkers would allow for the use of CDK inhibition in a more tailored manner, avoiding the drug and financial toxicities suggested by the SONIA trial. To find such biomarkers, innovations such as liquid biopsy, a blood-based method of profiling tumor-derived materials, are being employed. This method avoids the need for invasive biopsy, especially in metastatic lesions in which a tissue sample is required, as they often do not reflect the primary tumor biology [41].

### 4.1. Prediciting Response

De novo FAT1 mutations have shown potential to predict treatment resistance in ER+/HER2− advanced breast cancer. FAT1 is a member of the cadherin superfamily, which interacts with the Hippo signaling pathway. Interestingly, the loss of FAT1 is correlated with increased CDK6 mRNA levels mediated through the Hippo pathway. CDK6 amplification has been shown to be associated with CDK inhibitor resistance as well [42]. A study examined the effect of FAT1 mutations on PFS among 348 patients treated with palbociclib, ribociclib, and abemaciclib [43]. The pretreatment biopsies underwent genetic sequencing to determine the presence of FAT1. Patients with FAT1 mutations resulting in a loss of FAT1 had a significantly decreased median PFS of 2.4 months, compared to 10.1 months for patients without FAT1 mutations across all three CDK inhibitors [42]. It should be noted that de novo FAT1 loss-of-function mutations are only observed in approximately 6% of metastatic breast cancers [44]. This suggests that FAT1 mutations represent a small minority of patients who have primary resistance to CDK inhibitors.

The loss of RB1 determined by IHC has been studied extensively during the PALOMA-1 and -2 trials, which revealed that approximately 10% of patients developed acquired RB1 loss during treatment with palbociclib [3,4]. They found that those patients with RB1 loss had a statistically significant decrease in PFS compared to the RB1-positive patients of 3.6 months compared to 10.1 months. The PALOMA-3 trial studied RB1 loss via ctDNA and found that 5% of patients acquired RB1 loss with no significant impact on PFS [5]. Analysis of the ctDNA from patients in the MONALEESA-2, -3, and -7 trials found that for patients with RB1 mutations, ribociclib-plus-endocrine therapy did not significantly improve the median PFS [6,7,8]. Although this supports that RB1 loss can determine if a patient will respond to CDKi, given the small amount of RB loss mutations noted in these study populations, it appears that it only makes up a small fraction of the resistant mechanisms.

The PALOMA-1 trial also evaluated CCND1 and p16 ctDNA levels as potential markers; unfortunately, presence of these mutations did not correlate with changes in PFS [3]. Interestingly, a study evaluating patient-derived xenografts of ER+/HER2− early and advanced breast cancer showed that a high expression of the tumor suppressor p16 conferred de novo resistance to CDK inhibitors [45]. As mentioned earlier in this review, p27 has both activating and inhibiting properties on CDK 4/6. Its activating effects are dependent on phosphorylation at p27′s tyrosine-88 residue (pY88). One study found IHC staining of pY88 to be a biomarker predictive of de novo resistance, as pY88-negative tumor cells were resistant to palbociclib-mediated cell arrest, whereas pY88-positive patients were sensitive to it [46]. Taken together, it appears that when CD4/6 is heavily bound by either p16 or p27, it is resistant to inhibition by CDK inhibitors. This suggests that ctDNA levels of p16 or p27 could potentially predict CDK inhibitor responsiveness.

The PALOMA-3 trial also evaluated CCNE1 and CCNE2, the genes responsible for cyclin E, and found that the amplification of CCNE1/2 is associated with a poorer prognosis [5]. This coincides with preclinical models which showed that the over-expression of CCNE1 was found in CDK4/6 non-responders, and those with a low CCNE1 expression prior to treatment had a longer PFS [3]. Further, the MONALEESA-7 trial evaluated CCND1 and found that patients with CCND1 alterations in their baseline ctDNA had a worse median PFS for both treatment arms, indicating the role of CCND1 as a potential prognostic biomarker [8].

Mutations in other signaling pathways have also been studied as possible predictive biomarkers, such as PIK3CA, FGFR, TP53, and KRAS; however, none have been shown to be of significant clinical use.

The SONIA trial is currently comparing several biomarkers addressed above; however, the data are still immature.

### 4.2. Monitoring Response

The dynamic monitoring of ctDNA is being increasingly used to monitor treatment response in patients with cancer. For one, monitoring ctDNA levels at regular intervals provides information regarding the proliferative activity of the cancer. Monitoring ctDNA in this way can identify the ever-changing genetics and epigenetics within a cancer.

The ALCINA trial sought to determine if ctDNA can be used as a biomarker to determine treatment response with palbociclib and fulvestrant. A total of 25 patients in their study group were found to have somatic mutations in archived tissue that could be tracked by ctDNA. They took ctDNA samples at baseline, day 15, and day 30. They found that an elevated day-30-to-baseline ctDNA ratio was correlated with a worse PFS. Notably, ctDNA was present in all patients with radiologic progression, suggesting that serial ctDNA testing can anticipate radiologic progression. Baseline ctDNA did not correlate with PFS [47]. Given the small sample size, a larger study is needed to determine if serial ctDNA measurements are of clinical utility.

The PADA-1 trial successfully showed that biomarker monitoring can improve PFS. The PADA-1 trial, conducted in 83 French hospitals, investigated the efficacy and safety of adjusting treatment based on rising ESR1 mutations in blood (bESR1mut) for patients with advanced ER-positive, HER2-negative breast cancer. Of the 1017 enrolled women, 279 (27%) exhibited an increase in bESR1mut. Among these, 172 were randomized to either maintain their current aromatase inhibitor and palbociclib treatment or switch to fulvestrant and palbociclib. The results indicated that the group that switched to fulvestrant and palbociclib experienced a median progression-free survival of 11.9 months, which was significantly longer than the 5.7 months observed in the continued aromatase inhibitor group (HR 0.61; *p* 0.0040) [48]. This trial underscores the potential of the early therapeutic targeting of bESR1mut in providing significant clinical benefits and offers insights into addressing acquired resistance in future studies.

## 5. Conclusions

The advent of CDK inhibitors has dramatically shifted the paradigm of breast cancer treatment. Clinical data accumulated over the last decade convincingly demonstrate their efficacy, especially in HR+/HER2− breast cancer, heralding a new chapter in targeted therapies.

Concurrently, the emergence of ctDNA as a novel monitoring tool has provided profound insights into real-time tumor dynamics. ctDNA, due to its non-invasiveness and ability to capture tumor heterogeneity, is increasingly being recognized as a significant advancement in assessing treatment response. As CDK inhibition impacts the tumor milieu, changes in the ctDNA landscape can offer valuable insights into treatment efficacy and potentially forecast resistance mechanisms. By gauging these subtle genomic shifts in ctDNA, clinicians can now potentially tailor therapeutic strategies, making adaptive decisions during the treatment course rather than relying solely on empirical evidence.

In sum, the nexus between CDK inhibition and ctDNA monitoring embodies the epitome of precision oncology. As we delve deeper, it is hoped that we will usher in a new era of breast cancer management, one that is tailored to individual genetic landscapes and real-time tumor dynamics, ensuring the highest level of care for patients.

## Figures and Tables

**Table 1:** This table lists the CDK inhibitors along with their chemical structures and major toxicity profiles.

CDK Inhibitor	Chemical Structure	Adverse Effects
Palbociclib	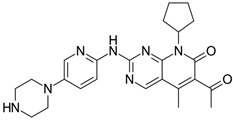	Myelosuppression
Ribociclib	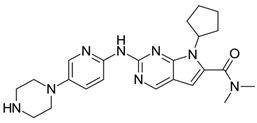	Myelosuppression QT prolongation
Abemaciclib	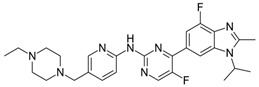	GI toxicity

**Table 2:** This table summarizes the seminal trials of the three FDA-approved cyclin-dependent kinase inhibitors for HR+/HER2− breast cancer.

Trial	N	Regimens	Median OS	Hazard Ratio (95% CI)	*p*-Value
PALOMA-3	521	Placebo + fulvestrant	28.0 months	0.81 (0.64–1.03)	0.09
		Palbociclib + fulvestrant	34.9 months		
MONALEESA-3	726	Placebo + fulvestrant	51.8 months	0.72 (0.57–0.92)	0.004
		Ribociclib + fulvestrant	67.6 months		
MONALEESA-7	672	Placebo + tamoxifen or NSAI	48.0 months	0.71 (0.53–0.95)	0.009
		Ribociclib + tamoxifen or NSAI	58.7 months		
MONARCH-2	669	Placebo + fulvestrant	37.3 months	0.76 (0.61–0.95)	0.01
		Abemaciclib + fulvestrant	46.7 months		

**Table 3:** This table summarizes the trials investigating the use of cyclin-dependent kinase inhibitors in the adjuvant setting.

Trial	Year	Status	Patient Population	Intervention	Control Arm	Primary Outcome
PENELOPE-B (NCT01864746)	2013–2020	Completed	HR+, HER2− EBC	PAL (125 mg once daily for 13 cycles) + ≥5 years ET	ET + placebo	42.8-month iDFS: no difference; (HR = 0.93, 95% CI: 0.74–1.17); two-sided weighted log-rank test (Cui, Hung, and Wang) *p* = 0.525
PALLAS (NCT02513394)	2015–2020	Completed	HR+, HER2− EBC	PAL (125 mg orally once daily for 2 years) + ≥5 years ET	ET alone	31-month iDFS: 8.8% PAL + ET vs. 9.1% ET, iDFS at 4 years: 84.2% vs. 84.5% (HR = 0.96, 95% CI: 0.81–1.14, *p* = 0.65)
MonarchE (NCT03155997)	2017–2020	Completed	HR+, HER2− EBC	Abemaciclib (150 mg twice daily for 2 years) + ET ≥5 years	ET alone	3-year iDFS improved by 5.4% in the abemaciclib group (HR = 0.70, 95% CI: 0.59–0.82)
MonarchE (NCT03155997)	2018–2026	Active	HR+, HER2− EBC	RIBO (400 mg/day 3 weeks on, 1 week off for 3 years) + ET ≥5 years	ET alone	34-month iDFS improved by 3.3% in the RIB + ET group; iDFS (HR, 0.748; 95% CI, 0.618–0.906; *p* = 0.0014); 3-year iDFS rates: 90.4% vs. 87.1%

**Table 4:** This table summarizes the trials investigating the use of cyclin-dependent kinase inhibitors in ER+/HER2+ breast cancer.

Trial	Phase	Setting	Arms	Primary Outcomes
PATINA Clinical study of the targeted therapy, palbociclib, to treat metastatic breast cancer (NCT02947685)	Phase III *n* = 496 International centers	Metastatic HR+/HER2+ breast cancer	Palbociclib + anti-HER2 therapy (trastuzumab/pertuzumab) + endocrine therapy (letrozole, anastrozole, exemestane, or fulvestrant) Control arm: anti-HER2 therapy (trastuzumab/pertuzumab) + endocrine therapy (letrozole, anastrozole, exemstane, or fulvestrant)	Progression-free survival (PFS)
PATRICIA II Palbociclib and trastuzumab with endocrine therapy in HER2-positive metastatic breast cancer (NCT02448420)	Phase II *n* = 102 Spain	Metastatic HR+/HER2+ breast cancer	Palbociclib + trastuzumab (HR-/HER2+) Palbociclib + trastuzumab (HR+/HER2+) Trastuzumab + palbociclib + letrozole (HR+/HER2+) Palbociclib, trastuzumab, and endocrine therapy (aromatase inhibitor, fulvestrant, or tamoxifen) for HR+/HER2+, luminal intrinsic subtype (PAM50) Control arm: physician’s choice (T-DM1 or chemotherapy (gemcitabine, vinorelbine, capecitabine, eribulin, or taxane) + trastuzumab or endocrine therapy + trastuzumab) for HR+/HER2+, luminal intrinsic subtype (PAM50)	Progression-free survival (PFS)
